# Pre-digestion of the lipids in infant formula affects gut maturation of the preterm pig

**DOI:** 10.1371/journal.pone.0265144

**Published:** 2022-03-16

**Authors:** Kamil Zaworski, Jarosław Woliński, Monika Słupecka-Ziemilska, Stefan Pierzynowski, Kateryna Pierzynowska

**Affiliations:** 1 Department of Animal Physiology, The Kielanowski Institute of Animal Nutrition and Physiology, Polish Academy of Sciences, Jablonna, Poland; 2 Department of Human Epigenetics, Mossakowski Medical Research Institute, Polish Academy of Sciences, Warsaw, Poland; 3 Department of Biology, Lund University, Lund, Sweden; 4 Department of Biology, Institute Rural Medicine, Lublin, Poland; 5 SGP + Group, Trelleborg, Sweden; Metrohealth Medical Center, UNITED STATES

## Abstract

Preterm birth is associated with increased risk of complications, specifically with regards to the gastrointestinal tract. These complications mainly include the maldigestion and malabsorption of nutrients resulting from the immaturity of the small intestine. The current study investigated whether pre-digestion of fat in infant formula would affect the developmental remodeling of the structure of the small intestine mucous membrane. Three groups of premature piglets (corresponding to 30–32 week of human gestation) were used in the study: the first group, not subjected to any treatment and euthanized within 2 hours after caesarian delivery, was used as the control group (PT group), the second group, was fed an infant formula—IF (SPT group), and the third group was fed a lipase pre-hydrolyzed infant formula—hIF (PPT group). Feeding preterm piglets with an infant formula for 14 days stimulated intestinal maturation (in SPT and PPT groups). However, pre-digestion of the infant formula with lipase significantly increased proliferative activity and intensity of apoptosis in the small intestine epithelium, resulting in more rapid enterocyte turnover. The data obtained not only confirm that starting enteral feeding directly after birth stimulates developmental and structural changes in the small intestine, but also highlighted the importance of lipid digestion for enterocyte turnover and speeding up of intestinal maturation in preterm piglets. The latest is of high importance for the proper gut development of preterm children.

## Introduction

Despite large advances in medicine in recent years, the number of preterm births continues to increase. Preterm birth is associated with multiple complications, such as infectious and non-infectious respiratory issues, necrotizing enterocolitis, neonatal jaundice, circulatory failure, retinopathy of prematurity, etc. These complications include functional immaturity of the intestinal functions, related to motility, digestion, and absorption [1].

Premature infants often demonstrate impaired digestion and absorption of nutrients from the lumen of the small intestine. These impairments are related to the immature intestinal structure, lack of exocrine pancreatic function and reduced bile synthesis and resorption [2]. Therefore, in preterm infants, the possibility of intestinal maturation stimulation, as well as effective pancreatic enzyme replacement therapy seems to be a crucial issue.

Piglets are often used in research investigating feeding methods and the influence of various bioactive substances on the condition of the intestine of preterm and full-term infants [3]. The morphology and physiology of the digestive tract of piglets is similar to that of preterm and neonatal human infants, including the rate of intestinal growth and maturation, the dimensions, weight and histological structure of the organs at birth. High similarities are also observed in the structure and physiology of the pancreas between piglets and human infants [4].

The intestinal endocrine system and the enteric nervous system are only partially developed at birth [5]. The biologically active ingredients within colostrum and mother’s milk not only provide a rapidly developing newborn with necessary nutrients, but also regulate the infant’s development, including the development of the digestive system. Biologically active ingredients within food affect the dynamics of the intestinal barrier development, show trophic activity in the intestine, and regulate the intensity of cell division in the intestinal crypts and the death of epithelial cells at the top of intestinal villi [6].

Mothers that give preterm birth, often experience an insufficient quantity of breast milk and difficulties with breast feeding. Donor milk which seems to be an acceptable alternative, should be pasteurized for the safety reasons [7]. Pasteurization destroys many of the bioactive milk components and affects casein structure [8]. Moreover, donor milk from mothers of full-term infants often does not meet the nutrition requirements of preterm infants, that’s why the fortification of donor milk is recommended [9]. The Holder pasteurization was suggested to be associated with impaired growth and development of preterm infants [10, 11], while the safety of donor milk fortification still remains controversial [12, 13]. Currently, milk formulas are used, the composition of which partially resembles that of mother’s milk [14]. Infant formulas contain fats, sugars, and proteins, as well as vitamins, and macro- and microelements [15]. It should be noted, however, that infant formulas have insufficient amounts of biologically active substances [16]. Parameters of intestinal maturation (weight, small intestine dimensions, intestinal crypt depth and villi length) have been found to be significantly improved in animals fed with mother’s milk compared to those fed with infant formula [17]. While new methods of donor milk enrichment and disinfection are under development, it is worth to pay more attention to preterm infant formulas, which could be customized and may be enriched in majority of the nutrients and bioactive compounds necessary for the premature infants’ growth and development.

Recent studies from our lab [18, 19] have shown that pre-digestion of infant formula with microbial lipase improved lipid absorption, tissue fat accretion and intestinal structure in young pigs with exocrine pancreatic insufficiency. The present study aimed to investigate whether pre-digestion of the lipids in infant formula could affect intestinal remodeling and improve intestinal structure in a porcine model of the premature human infant.

## Materials and methods

### Animals

The present study was performed in strict accordance with the recommendations of the Guide for the Care and Use of Laboratory Animals of the National Institutes of Health. All efforts were made to minimize animal suffering. The study was approved by the Second Local Ethics Committee for Animal Experimentation in Warsaw, Poland (approval no. WAW2_8/2016).

The experiments were performed on 22 piglets (Polish synthetic line 990) delivered by caesarean section, from two sows inseminated by the same boar semen, 8 days before expected labor (107 days, 93% gestation, corresponding to 30–32 weeks human gestation). Piglets were housed in individual cages (1 x 1.5 m), equipped with red heating lamps (150 W, developing temp. at cage 36–38°C, humidity ~60%) on a 12-hour day-night cycle, with lights on from 06.00–18.00 (6 am-6 pm). In order to ensure the proper development and survival of the preterm piglets immediately after delivery, they were infused with porcine immunoglobulins (Ig) (1540 mg/kg bwt—25 ml of sterile Ig preparation/kg bwt) via the umbilical artery as previously described [20, 21]. Porcine immunoglobulin (IG) preparation was obtained from a pool of blood plasma obtained from two multiparous sows by ammonium sulphate precipitation [22]. The IG formulation was sterile filtered and stored at −20 °C until use. Gastric ports and jugular vein catheters were implanted during a surgical procedure that took place after caesarean section (during the first 12 hours of the animal’s postnatal life [20, 21]). After surgery, all piglets received a 5% glucose infusion via the jugular vein catheter, at a rate of ~3 mL/h, for a period of 8 hours. Enteral feeding via the stomach port was initiated immediately following the first initial 8-hour period after surgery. All piglets were fed human infant formula (IF) (10 ml/kg bwt; Similac Special Care 24, Abbott Nutrition, Columbus, Ohio, USA, composition is provided in the Table 1) via a gastric port, every 2 hours.

**Table 1:** Composition of Similac Special Care 24 nutritional formula, manufactured by Abbott Nutrition, USA (https://abbottnutrition.com/similac-special-care-24).

Nutrient Data	Amount per Serving
Calories	100
Volume, ml	123.22
Protein, g	3.00
Fat, g	5.43
Carbohydrate, g	10.30
Water, g	109
Linoleic Acid, mg	700
Potential Renal Solute Load, mOsm	27.8
**Vitamins**	
Vitamin A, IU	1250
Vitamin D, IU	150
Vitamin E, IU	4
Vitamin K, mcg	12
Thiamin (Vitamin B1), mcg	250
Riboflavin (Vitamin B2), mcg	620
Vitamin B6, mcg	250
Vitamin B12, mcg	0.55
Niacin, mcg	5000
Folic Acid (Folacin), mcg	37
Pantothenic Acid, mcg	1900
Biotin, mcg	37
Vitamin C (Ascorbic Acid), mg	37
Choline, mg	10
Inositol, mg	40
**Minerals**	
Calcium, mg	180
Phosphorus, mg	100
Magnesium, mg	12
Iron, mg	1.8
Zinc, mg	1.5
Manganese, mcg	12
Copper, mcg	250
Iodine, mcg	6
Selenium, mcg	2
Sodium, mg	43
Potassium, mg	129
Chloride, mg	81

Water, Nonfat Milk, Corn Syrup Solids, Medium chain Triglycerides, Lactose, Whey Protein Concentrate, Soy Oil, Coconut Oil. Less than 0.5% of: C. Cohnii Oil, M. Alpina Oil, Beta-Carotene, Lutein, Calcium Phosphate, Ascorbic Acid, Potassium Citrate, Calcium Carbonate, Soy Lecithin, Monoglycerides, Magnesium Chloride, m-Inositol, Sodium Citrate, Carrageenan, Potassium Hydroxide, Ferrous Sulfate, Choline Bitartrate, Taurine, Choline Chloride, Niacinamide, L-Carnitine, Zinc Sulfate, Potassium Chloride, Salt, Potassium Phosphate, d-Alpha-Tocopheryl Acetate, Calcium Pantothenate, Vitamin A Palmitate, Cupric Sulfate, Riboflavin, Thiamine Chloride Hydrochloride, Pyridoxine Hydrochloride, Folic Acid, Manganese Sulfate, Biotin, Phylloquinone, Sodium Selenate, Vitamin D3, Cyanocobalamin, and Nucleotides (Cytidine 5’-Monophosphate, Disodium Guanosine 5’-Monophosphate, Disodium Uridine 5’-Monophosphate, Adenosine 5’-Monophosphate).

### Experimental design

**Fig 1:**
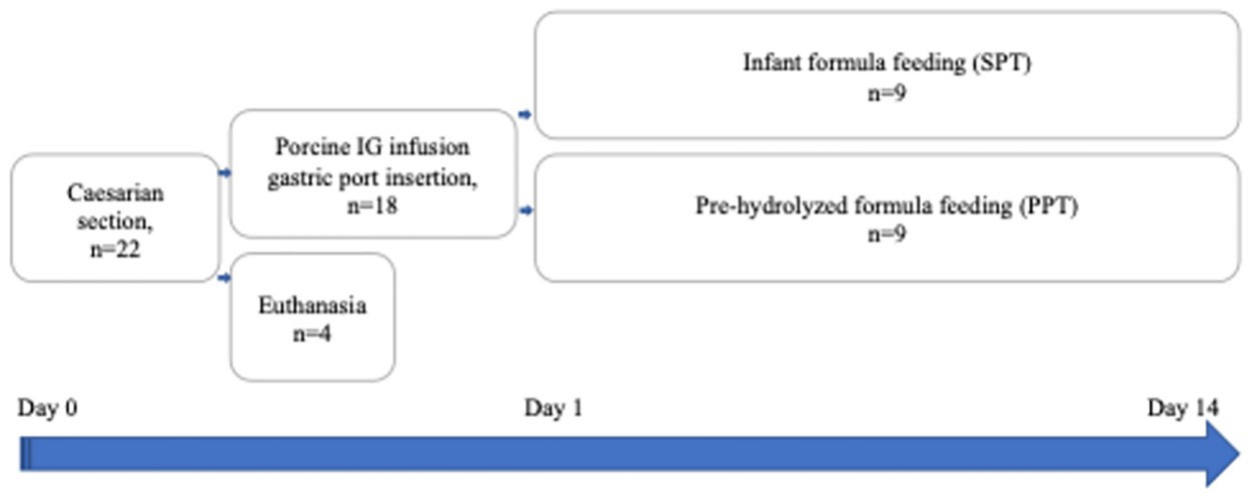
Schematic representation of the study design. IG—immunoglobulins, SPT—preterm piglets fed infant formula; PPT—preterm piglets fed pre-hydrolyzed infant formula.

Sample size was estimated using G*Power software, version 3.1.9.4 [23] for a one-way ANOVA at α = 0.05 with 95% power, assuming f (effect size) = 1.5 and SD = 20, for three study groups. The calculation yielded a total sample size of 12, resulting in 4 animals per treatment group. A schematic representation of the study design is shown in Fig 1. After gastric port insertion, piglets were randomly divided into 2 groups depending on treatment: preterm piglets (n = 9) fed infant formula (IF) (SPT group); preterm piglets (n = 9) fed pre-hydrolyzed IF (PPT group); preterm piglets (n = 4) not subjected to any treatment and euthanized within 2 hours after delivery served as an intact, control group (PT group). The infant formula fed to the PPT group was hydrolyzed as previously described [19, 24]. In brief, immobilized microbial lipase (iML, Lipase 534641, Sigma-Aldrich) in a mesh bag was placed in the infant formula mixture and stirred for 15 minutes at 35–37°C before each feeding.

The experimental period continued for the next 13 days. On the 14^th^ day of the experiment, the pigs were euthanized by intravenous injection of sodium pentobarbiturate (100 mg/kg) (Morbital; Biowet, Puławy, Poland).

### Histomorphometric analysis

At dissection, samples collected from the small intestine: proximal, middle and distal part of jejunum, were fixed in 10% neutral formalin solution fixative for 24 hours. Prior to the embedding, cross-sectional tissue samples containing all structural layers were dissected out from a 3 cm long sample (approximately 5 mm). Next, tissue pieces were dehydrated and embedded in paraffin according to standard histological technique [25]. Paraffin-embedded tissues were sliced using rotor microtome into sections 4.5 μm thick. After overnight drying, slides were deparaffinized by incubation in xylene and decreasing concentrations of ethanol. Finally, standard hematoxylin and eosin staining was performed [26]. Analysis was performed using a light microscope (Axioskop 40, Zeiss, Germany), equipped with a digital camera (Coolpix B700, Nikon). The obtained data was accessed with an Axio Vision software, version 4.2 (Zeiss, Germany). Parameters such as lamina muscularis and mucosa thickness, villus length and crypt depth were measured. The number and area of lysosomal vacuoles (LV) were estimated as well. For each section, a minimum of 20 measurements of each of the parameters were made.

### Immunohistochemical analysis

#### Intestinal crypt stem cell proliferative activity

For immunohistochemical analysis, sections were dehydrated in alcohol series and washed with 0.1M PBS (PBS tablets, Medicago, Sweden). The sections were then placed into boiling citrate buffer (10 mM citric acid, 0,05% Tween 20, pH 6.0) for 10 min, washed with 0.1M PBS and incubated for five min in 0,3% solution of H_2_O_2_. The sections were then rinsed with 0.1M PBS and primary antibodies to Ki67 (marker of proliferation) (rabbit polyclonal antibodies to Ki67; Sigma Aldrich, Germany) were applied in a dilution of 1:500. After 30 min of incubation in a humid chamber at room temperature, sections were rinsed twice with 0.1M PBS and incubated in PBS for 5 min during the last rinsing. Rabbit-specific HRP polymer (Envision+, Dako, Denmark) was used for primary antibodies detection. Sections were incubated for 30 min in a humid chamber at room temperature and then washed twice with 0.1M PBS. Following this step, the 3,3′-diaminobenzidine (DAB, Envision+, Dako, Denmark) substrate was applied and sections were incubated for 8 min and then rinsed with distilled water and stained with hematoxylin for nuclei visualization. Sections were mounted with Paramount (catalogue number S3025, Dako, Denmark) and the mitotic index, as the percentage of Ki67 positive cells (3,3′-diaminobenzidine staining, brown) among all epithelial cells in the crypt cross-section, was calculated. For each section, a minimum of 20 measurements were made under a light microscope (Axioskop 40, Zeiss, Germany, magnification—400×).

#### Apoptotic index of the epithelial cells of the small intestine

An ApopTag^®^ Red In Situ Apoptosis Detection Kit (S7165, Chemicon, USA) was used to detect and label apoptotic cells in the epithelium of the small intestine. The sections were placed into boiling citrate buffer (10 mM citric acid, 0,05% Tween 20, pH 6.0) for 10 min and then washed with 0.1M PBS. Then the procedure recommended by the manufacturer was followed. Sections were mounted with Fluorescent Mounting Medium (catalogue number S3023, DakoCytomation, Denmark). The apoptotic index was assessed as the ratio of apoptotic cells to the total number of epithelial cells of the villi (1/3 of the villi length from the villi apex). For each section a minimum of 20 measurements were made, the tissue images were analyzed using a confocal microscope (LSM 5 PASCAL, Zeiss, Germany).

### Statistical analysis

Data are expressed as mean ± SD (when normally distributed) or median ± IQR (when distribution was nonparametric). Normally distributed data was assessed using an ANOVA, followed by a Tukey post hoc test and datasets with nonparametric distribution were assessed using a Kruskal-Wallis test followed by a Dunn’s multiple comparison test (GraphPad Prism, v 8.1.0, San Diego, CA, USA). To assess data distribution, a Shapiro-Wilk normality test was performed. Differences were considered significant if p ≤ 0:05.

## Results

### Histomorphometry analysis

**Fig 2:**
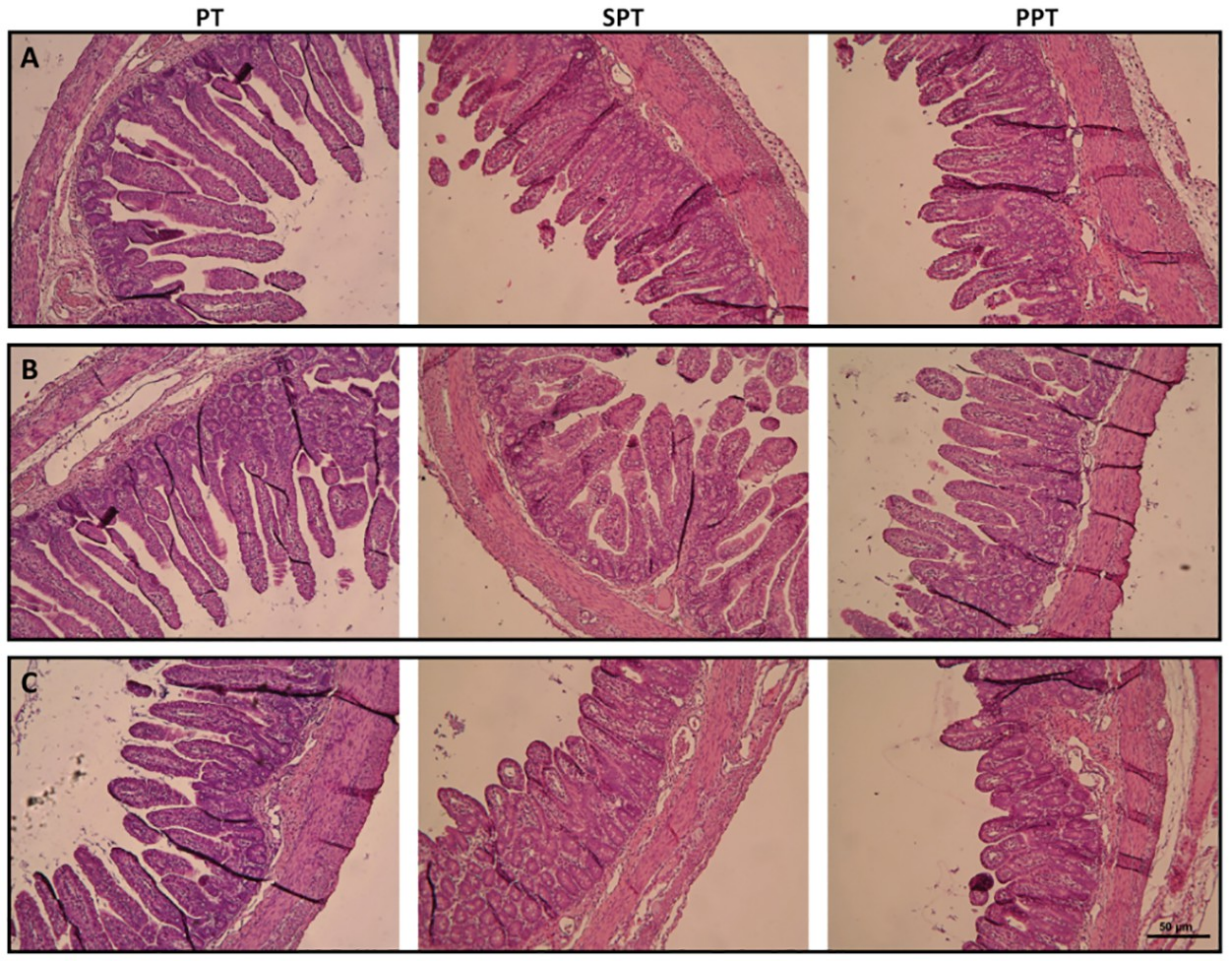
Morphological changes of small intestine (jejunum). PT—14-day old intact, control preterm piglets, SPT—preterm piglets fed infant formula, PPT—preterm piglets fed pre-hydrolyzed infant formula. A—proximal, B—middle, C—distal, section of the jejunum. Images were obtained using a light microscope (Axioskop 40, Zeiss, Germany, magnification—400×).

Analysis of the jejunum segments of piglets fed infant formula (SPT group) and pre-hydrolyzed infant formula (PPT group) revealed a significant decrease in mucosa thickness, villus length and crypt depth, in comparison to the control group of intact, preterm piglets (PT group) in all jejunal segments (proximal, middle, distal) (Fig 2 and Table 2). In the middle segment of the jejunum, a significant reduction in muscularis thickness was also observed in these two experimental groups (SPT and PPT) in comparison to control one (PT group). There were no significant differences between SPT and PPT groups (Table 2).

**Table 2:** Histomorphometric parameters measured in the proximal, middle, and distal segments of jejunum in preterm piglets.

Parameter/Group	PT	SPT	PPT
Proximal			
Mucosa thickness, μm	870.85 ± 176.82^a^	304.23 ± 91.20^b^	271.48 ± 61.83^b^
Villi length, μm	775.15 ± 162.71^a^	218.19 ± 73.95^b^	195.62 ± 40.00^b^
Crypt depth, μm	99.97 ± 15.71^a^	63.23 ± 18.35^b^	50.78 ± 24.04^b^
Muscularis thickness, μm	59.33 ± 12.54	42.68 ± 19.14	31.83 ± 19.15
Middle			
Mucosa thickness, μm	865.82 ± 153.37^a^	251.72 ± 76.02^b^	303.35 ± 39.60^b^
Villi length, μm	773.57 ± 149.24^a^	186.90 ± 60.05^b^	220.16 ± 41.55^b^
Crypt depth, μm	88.75 ± 13.54^a^	47.50 ± 9.89^b^	55.81 ± 14.52^b^
Muscularis thickness, μm	81.37 ± 16.68^a^	40.90 ± 7.16^b^	46.78 ± 6.38^b^
Distal			
Mucosa thickness, μm	546.30 ± 64.10^a^	254.95 ± 54.83^b^	287.54 ± 87.88^b^
Villi length, μm	530.53 ± 73.28^a^	159.91 ± 34.77^b^	203.31 ± 50.00^b^
Crypt depth, μm	94.02 ± 15.91^a^	62.66 ± 18.30^b^	51.48 ± 17.36^b^
Muscularis thickness, μm	58.86 ± 16.09	46.08 ± 13.79	43.89 ± 17.63

Preterm piglets without treatment—PT, preterm piglets fed infant formula—SPT, and preterm piglets fed pre-hydrolyzed infant formula—PPT. Values are given as Mean ± SD. Data was assessed using an ANOVA, followed by a Tukey post hoc test.

^a,b,c^ Values within a row with different superscript letters differ significantly at *p* < 0.05.

Regarding the lysosomal vacuoles (LV), no differences were observed in the proximal segment of jejunum, while in the middle segment the analysis revealed a significant (p<0.05) increase both in the number and common area of LV in both SPT and PPT groups (Table 3). In the distal segment, despite significant (p<0.05) reduction in LV number observed in both SPT and PPT groups, when compared to the PT group, the common LV area was not changed (Table 3).

**Table 3:** Parameters of lysosomal vacuoles (LV) measured in the proximal, middle and distal segments of the small intestine in preterm piglets.

Parameter/Group	PT	SPT	PPT
Proximal			
Number of LV per villi	2.72 ± 5.43	0.43 ± 2.57	2.29 ± 34.00
% of enterocytes with LV	2.81 ± 6.14	0.60 ± 4.15	2.89 ± 36.96
LV Area, μm^2^	80.31 ± 65.42	52.59 ± 82.10	54.11 ± 79.74
Middle			
Number of LV per villi	0.00 ± 0.17^a^	1.00 ± 4.15^b^	2.00 ± 2.43^b^
% of enterocytes with LV	0.00 ± 0.13^a^	1.00 ± 4.15^b^	2.33 ±113.00^b^
LV Area, μm^2^	0.00 ± 19.66^a^	46.09 ± 39.27^b^	67.58 ± 68.00^b^
Distal			
Number of LV per villi	75.42 ± 96.00^a^	3.29 ± 15.40^b^	11.71 ± 30.93^b^
% of enterocytes with LV	90.01 ± 76.01^a^	4.29 ± 18.54^b^	7.60 ±33.13^b^
LV Area, μm^2^	68.96 ± 33.60	65.60± 60.58	54.08 ± 40.55

Preterm piglets without treatment—PT, preterm piglets fed infant formula—SPT, and preterm piglets fed pre-hydrolyzed infant formula—PPT, LV—lysosomal vacuoles. Values are given as Median ± IQR.

^a,b,c^ Values within a row with different superscript letters differ significantly at *p* < 0.05.

Data was assessed using a Kruskal-Wallis test followed by a Dunn’s multiple comparison test.

### Intestinal crypt stem cells proliferative activity

**Fig 3:**
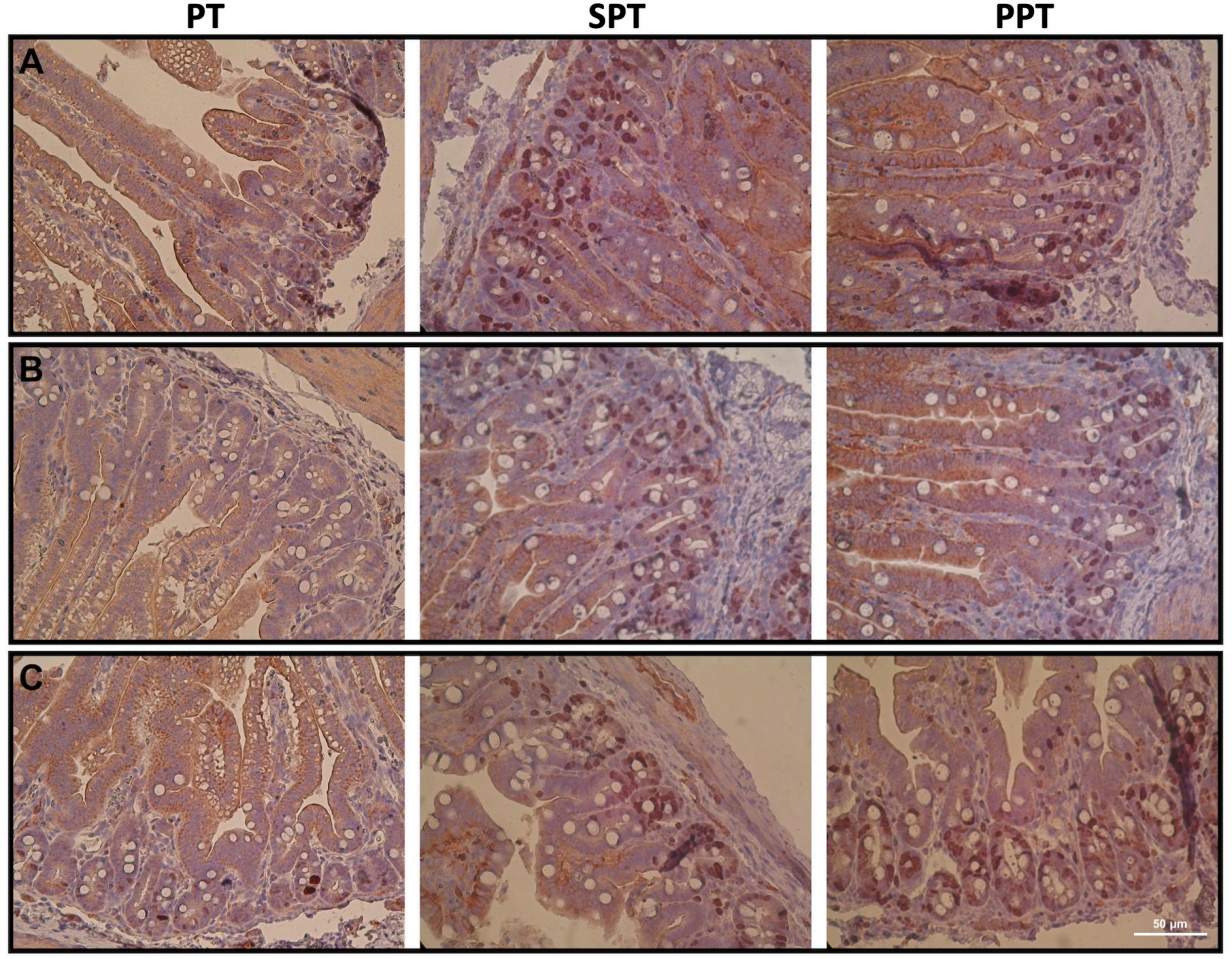
Changes in intestinal crypt stem cell proliferative activity. PT—14-day old intact, control preterm piglets, SPT—preterm piglets fed infant formula, PPT—preterm piglets fed pre-hydrolyzed infant formula. The images show stained Ki67 positive cells (3,3′-diaminobenzidine staining, colour—brown) prone to mitotic division. A—proximal, B—middle, C—distal, section of the jejunum. Hematoxylin-stained cell nucleus (colour—blue). Images were obtained using a light microscope (Axioskop 40, Zeiss, Germany, magnification—400×).

In all segments of the jejunum, a significant (p<0.0001) increase in proliferative activity was observed after 14 days of treatment in both groups of piglets (SPT and PPT), when compared to the control intact piglets (PT group). At the same time, piglets fed pre-hydrolyzed infant formula (PPT group) demonstrated the highest mitotic index in the proximal and distal segments (p<0.0001) of the jejunum. However, no differences in mitotic index of the middle jejunum segment were observed between piglets from the SPT and PPT groups (Fig 3 and Table 4).

**Table 4:** Proliferative activity of stem cells of the intestinal crypts of the intestinal mucosa epithelium (KI—Ki67 positive cells) and apoptosis of intestinal villi epithelial cells in the mucosa (AC—apoptotic cells) in the proximal, middle, and distal segments of the small intestine in preterm piglets.

		PT	SPT	PPT
Proximal	KI	2.00 ± 3.00^a^	6.00 ± 20.00^b^	9.00 ± 21.00^c^
	AC	1.00 ± 1.00^a^	5.00 ± 24.00^b^	4.00 ± 22.00^b^
Middle	KI	2.00 ± 3.00^a^	9.00 ± 20.00^b^	9.00 ± 24.00^b^
	AC	1.00 ± 3.00^a^	3.00 ± 12.00^b^	4.00 ± 10.00^b^
Distal	KI	1.00 ± 2.00^a^	5.00 ± 26.00^b^	7.50 ± 18.00^c^
	AC	7.50 ±12.00^a^	3.00 ± 8.00^b^	4.00 ± 21.00^a^

Preterm piglets without treatment—PT, preterm piglets fed infant formula—SPT, and preterm piglets fed pre-hydrolyzed infant formula—PPT, KI—Ki67 positive cells; AC—apoptotic cells. Values are given as Median ± IQR

^a,b,c^ Values within a row with different superscript letters differ significantly at *p* < 0.05.

Data was assessed using a Kruskal-Wallis test followed by a Dunn’s multiple comparison test.

### Apoptosis in intestinal villi epithelial cells

**Fig 4:**
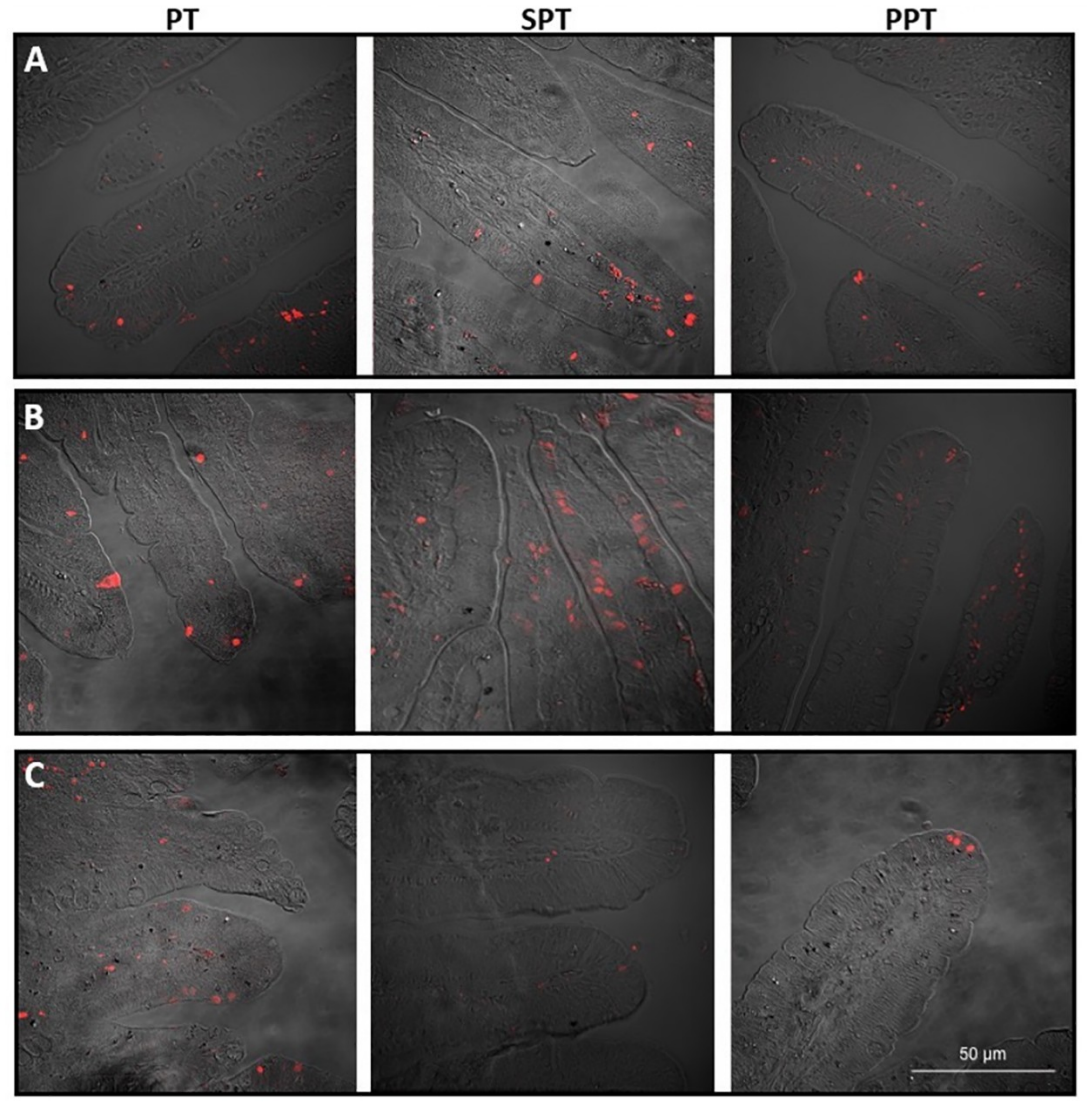
Apoptotic activity in the small intestinal villi. PT—14-day old intact, control preterm piglets, SPT—preterm piglets fed infant formula, PPT—preterm piglets fed pre-hydrolyzed infant formula. The images show visible apoptotic cells (colour—red) labeled with terminal deoxynucleotidyl transferase-mediated deoxyuridine triphosphate nick end labeling assay. A—proximal, B—middle, C—distal, section of the jejunum. Images were obtained using a confocal microscope (LSM5 Pascal, Zeiss, Germany, magnification—400×).

In the proximal and middle segments of the jejunum, a statistically significant (p<0.0001 for proximal and p = 0.01 for the middle segment) increase in apoptosis of the villus epithelial cells was observed after 14 days of treatment in both the SPT and PPT groups of piglets, when compared to the intact, control ones (PT group). At the same time, the apoptotic index of the distal segment of the jejunum was significantly (p<0.05) decreased in the SPT group in comparison to both PT and PPT groups (Fig 4 and Table 4).

## Discussion

The purpose of this study was to determine the effects of the pre-hydrolysis of the lipids in infant formula before feeding on the maturation of the small intestine in immunoglobulin-treated premature piglets. Preterm piglets delivered by caesarean section and then euthanized immediately after birth were used as a negative control.

Because the epitheliochorial placenta of the pig, in contrast to the human hemochorial placenta, does not allow the prenatal transfer of maternal immunoglobulins, all piglets are born with agammaglobulinemia. Thus, both to ensure adequate growth of the animals and to better mimic situation in preterm infants, preterm piglets which are used as a model of human neonates need to be supported with the porcine immunoglobulins [20, 27].

Premature labor affects the prenatal maturation of organs, including the gastrointestinal tract, and increases the risk of many diseases. Necrotizing enterocolitis (NEC), which is closely related to gastrointestinal immaturity, enteral nutrition and bacterial colonization can serve as an example. Infants with NEC may require resection of necrotic parts of the intestine [28, 29].

NEC is often observed in formula-fed preterm piglets [30, 31], however, upon physical examination, preterm piglets in the described study showed no clinical signs of NEC (e.g., abdominal distention, vomiting, diarrhea, bloody stools, feed retention, fever, etc.). These observations are in line with our previous reports [20, 21]. The important feature of the used porcine model is the intravenous infusion of purified porcine immunoglobulins, which allows the administration of immunoglobulins in sufficient amounts as it was described previously [20, 21]. This infusion was shown to have a considerable impact on the NEC prevention, providing a possibility to analyze ‘physiological’ intestinal maturation and possible nutritional impacts.

Due to the fact that the preterm infant’s digestive tract is not fully developed, it was assumed that the pre-hydrolysis process would increase the bioavailability of fats, since the natural hydrolysis of lipids by pancreatic lipases is rather limited [32]. Also, it was hypothesized that pre-hydrolyzed IF have a beneficial effect on the remodeling and maturation of the mucosa of the small intestine in the premature piglets. Previous studies using porcine models have shown that the most intensive development of the small intestine in newborn piglets occurs during the first 7 days of life [33]. The dynamics of small intestinal epithelial remodeling in premature piglets is still not completely understood. The time of the presented experiment (14 days) was associated with the assumption that particularly during this time a low pancreatic secretory capacity is typically observed in both premature infants and other newborn mammals [34]. Sangild et al. [31] used a similar experimental period duration in their study in which they investigated the effects of bovine colostrum and human donor milk on gut development, immune system development and intestinal bacterial colonization of premature piglets. The experiments were carried out for 11 days from the moment of preterm labor and significant changes in most of the parameters tested were observed.

The results of the histomorphometry and immunohistochemical analysis of the small intestine in intact, control preterm pigs in the current study highlight the specificity of the changes occurring in the intestine during the early postnatal period. In the group of intact, control preterm piglets (PT), an increased mucosa thickness, villus length and crypt depth were noted in all sections of the small intestine assessed in comparison to both groups of piglets fed infant formula (SPT and PPT).

It is assumed that the mucosal hypertrophy observed in the suckling piglets is associated with the presence of fetal enterocytes, the size of which, due to the presence of lysosomal vacuoles, is much larger than that of so-called mature enterocytes. The number of lysosomal vacuoles and their area were significantly increased in the middle segment of jejunum in both SPT and PPT group, while in the distal segment the reduction in number of these vacuoles and vacuolated enterocytes, but not in the total area of vacuoles, appeared (when compared to PT group). From studies on full-term piglets, it is known that transport vacuoles participating in macromolecule passage to the blood disappear after 2–3 days after delivery [35, 36]. Digestive vacuoles are able to uptake the intestinal content into enterocytes and digest it there. They are present in the small intestine of newborn piglets up to 3–4 weeks of postnatal life [35–37]. Both types of vacuoles disappear gradually. The dynamics of vacuolated enterocytes’ development is well studied for full-term pigs [38] and even for pigs with intrauterine growth retardation [39], but it still requires to be thoroughly investigated in pre-term pigs. Our data suggest the presence of mechanisms upregulating the development of digestive vacuoles in the middle part of jejunum, which are stimulated by enteral feeding and most probably aim to increase nutrients digestibility in preterm piglets. Even in the distal segment of jejunum, the total area of lysosomal vacuoles does not change despite the reduction of vacuolated enterocytes’ number, allowing better and prolonged availability of intestinal content for digestion.

A significant increase in the proliferative activity and the intensity of the apoptosis process in the epithelium of the small intestine was observed in piglets fed infant formula (SPT and PPT groups), in comparison to the intact, control preterm piglets (PT). These results demonstrate that, in comparison to the intact, control preterm pigs (PT), the enteral administration of the mixture alone stimulates growth and maturation of the small intestine (i.e., closing the intestinal barrier—this can be partially due to immunoglobulin infusion and replacement of the fetal enterocyte population with mature enterocytes). Similar characteristics of changes in the intestinal mucosa after administration of the mixture were observed in a piglet model used by Blättler et al. [40] and Biernat et al. [41], additionally indicating a change in the shape of the intestinal villi in the following days of life.

The data obtained in the current study demonstrates that feeding premature, newborn piglets pre-hydrolyzed IF does not cause any differences in mucosa thickness, villus length and crypt depth in all examined sections of the small intestine in comparison to the piglets fed pure IF. However, at the same time, in the group of piglets fed pre-hydrolyzed formula (PPT), an increase in the proliferative activity of intestinal crypt stem cells in the proximal and distal segments of the small intestine and an increase in the number of cells undergoing apoptosis at the tips of the intestinal villi in the proximal segment of the small intestine were observed, in comparison to that in the pure formula-fed piglets (SPT group). The possible explanation could be that the hydrolysis of lipids, and its products, stimulate among others GSK-3β/β-catenin signaling pathway. This causes the division of stem cells of the intestinal crypts, accelerates their migration and affects the viability of intestinal epithelial cells, resulting in the overall enhancement of enterocyte turnover [42].

Our results also show that the distal segment of the jejunum is the most sensitive to IF and nutrient availability. The villus length and crypts depth (i.e., the intestinal lymphatic surface) were the smallest in the distal part of the small intestine of the piglets fed the non-hydrolyzed formula, in comparison to other small intestine sections. It is also worth mentioning that the distal segment of the small intestine is the one that matures last, as manifested by the disappearance of fetal enterocytes. It seems that these factors made the distal segment of the small intestine most sensitive to the nutrient form and availability in the formula mixture after the hydrolysis process e.g., enterocyte apoptosis was the strongest in PPT pigs.

Of course, our results should be interpreted with care, as the main limitation of our study is the difference between porcine and human digestive system (differences in developmental patterns of intestinal maturation and carbohydrate digestion, enzyme profiles, different length and absorption area of small intestine, strong dependency of intestinal metabolism on amino acids in pigs, differences in intestinal trophic response, etc.). One should remember that the piglet gastrointestinal tract is probably even more premature than that of human infants born at similar gestational terms. Moreover, the mechanisms underlying the observed effects require thorough further investigation. However, the patterns of mucosal maturation, cell differentiation, and development of tissue architecture from proximal to distal regions of small intestine are very similar [43] and preterm piglet is well recognized as a relevant model to study intestinal structure, as well as nutrients’ digestion and absorption in premature human infants.

To our knowledge, the current work is the first one not only confirming that starting enteral feeding directly after birth stimulates in physiological manner the developmental and structural changes in the small intestine, but also showing the importance of lipid digestion not only for further lipids’ absorption *per se*, but also for enterocyte turnover and intensification of intestinal maturation in the small intestine in the model of porcine immunoglobulins-treated preterm piglet.
